# Supplementary material for: Phase-dependent amplification of working memory content and performance
Source: Nat Commun. 2020 Apr 14;11:1832. doi: 10.1038/s41467-020-15629-7 (PMC7156664; doi:10.1038/s41467-020-15629-7)
Supplement: Supplementary file 1 — Supplementary Information [file 41467_2020_15629_MOESM1_ESM.pdf]

Supplementary information

Phase-dependent amplification of working memory content and performance

Ten Oever et al.

Supplementary Figures

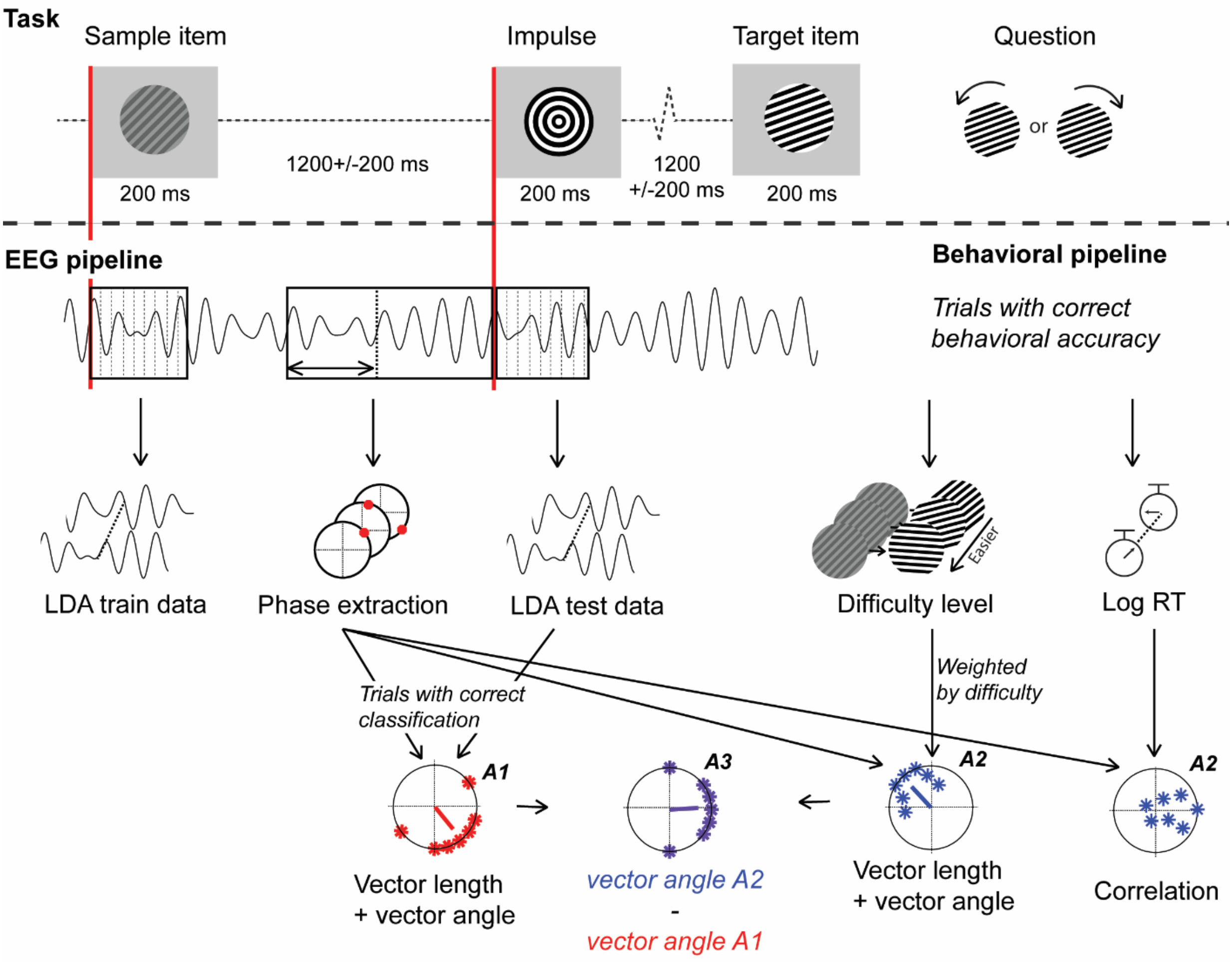

Supplementary Figure 1. Task and EEG pipeline. Training data for the LDA analysis was extracted from the EEG at time points directly after sample item presentation. Testing data was extracted from EEG data directly after impulse presentation. Phases were extracted for frequencies ranging between 4-12 Hz using data three cycles prior to impulse onset until impulse onset. For correctly classified trials the vector angle and vector length of the phases were extracted (A1, corresponding to data in Figure 1A). For the behavior analysis we extracted trials with correct performance (for all trials at a difficulty level of 50-75% accuracy) and again calculated the vector length and vector angle (weighted by difficulty). For reaction time we performed a correlation between phase and the logarithm of reaction times (A2, corresponding to data in Figure 2A). Lastly, we compared the angle from A1 and A2 by calculating their difference and comparing its difference to zero (A3, corresponding to data in Figure 2C).

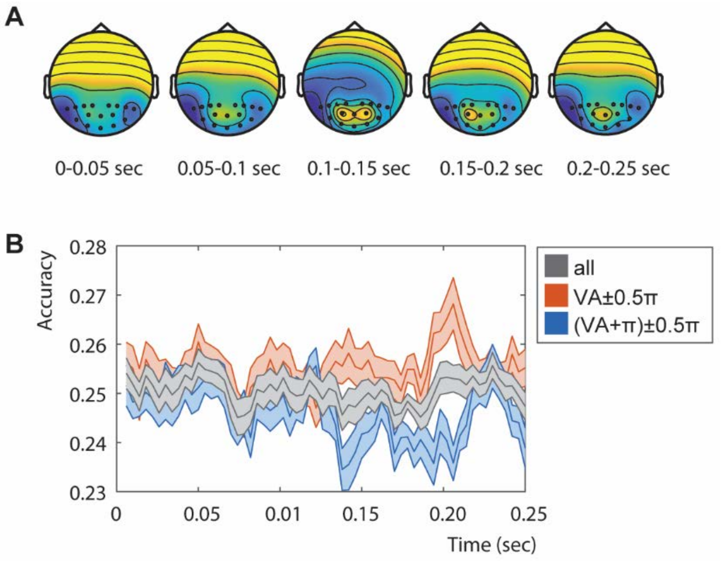

Supplementary Figure 2. Classifier performance. A) The weights of the classifier over time for all of the included channels. B) Time course of the classification performance for all trials, trials at the vector angle (VA) and trials around VA + π for the frequency and time point of the maximum VL-value. Shaded areas show the standard error of the mean (n=19).

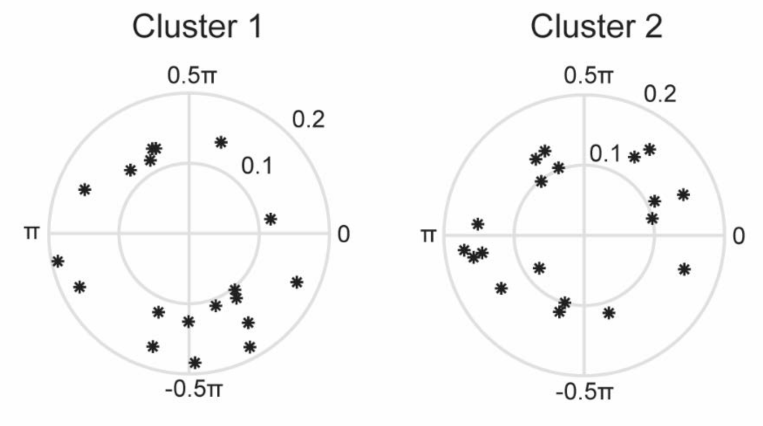

Supplementary Figure 3. Individual vector lengths and directions. Asterisk indicate the individual vector angle and vector length (radius).

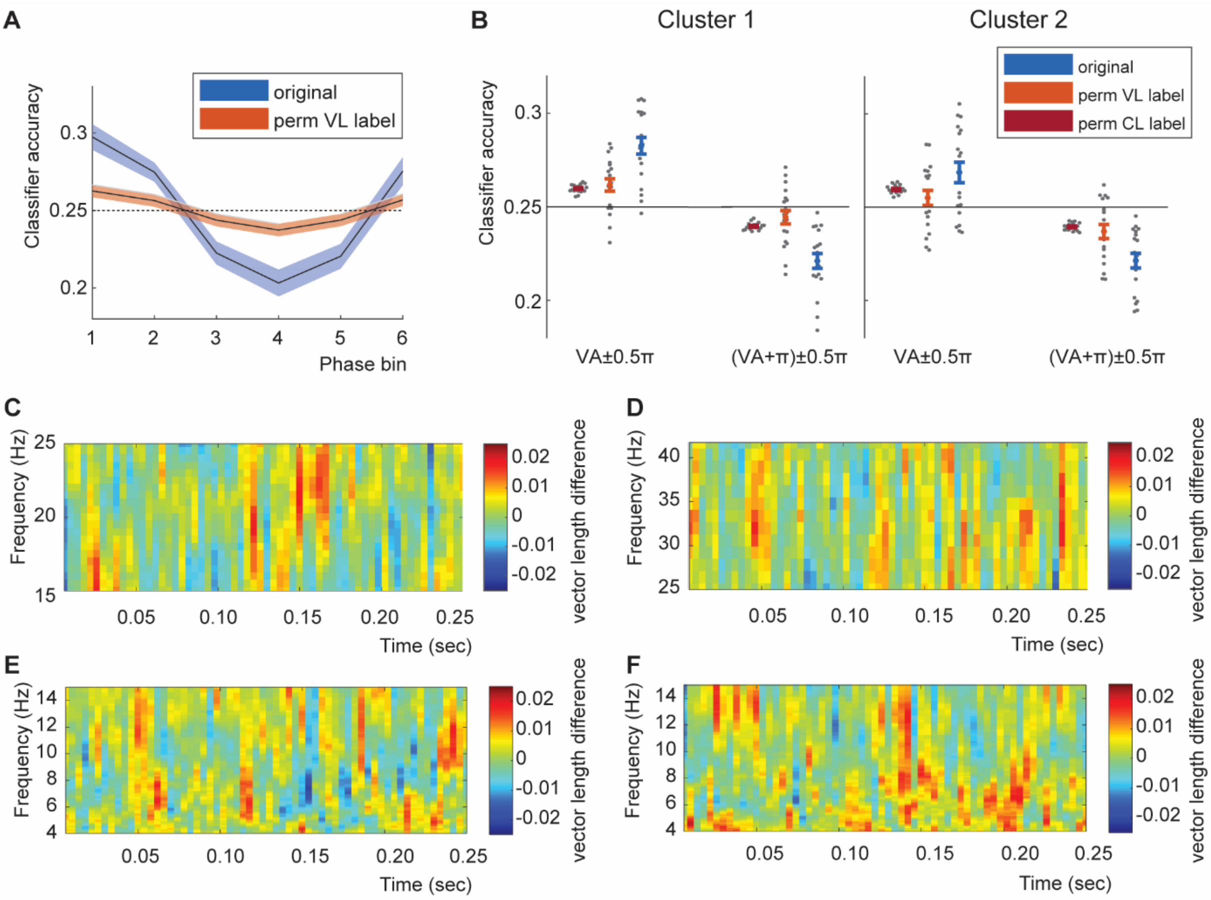

Supplementary Figure 4. Expanded phase modulation of decoding accuracy. A) Comparison of classifier accuracy for six equally spaced phase bins with VL label permutations for the max at each cluster (n=19). B) Classification accuracy for the two clusters separately. Error bars represent the standard error of the mean (n=19). No difference was found. Conventions of B are the same as in Figure 1B. C+D) Vector length difference for a wider beta and gamma cluster. No significant effects were found. E+F) Vector length difference for using only the frontal channels € or all channels (F). No significant effects were found.

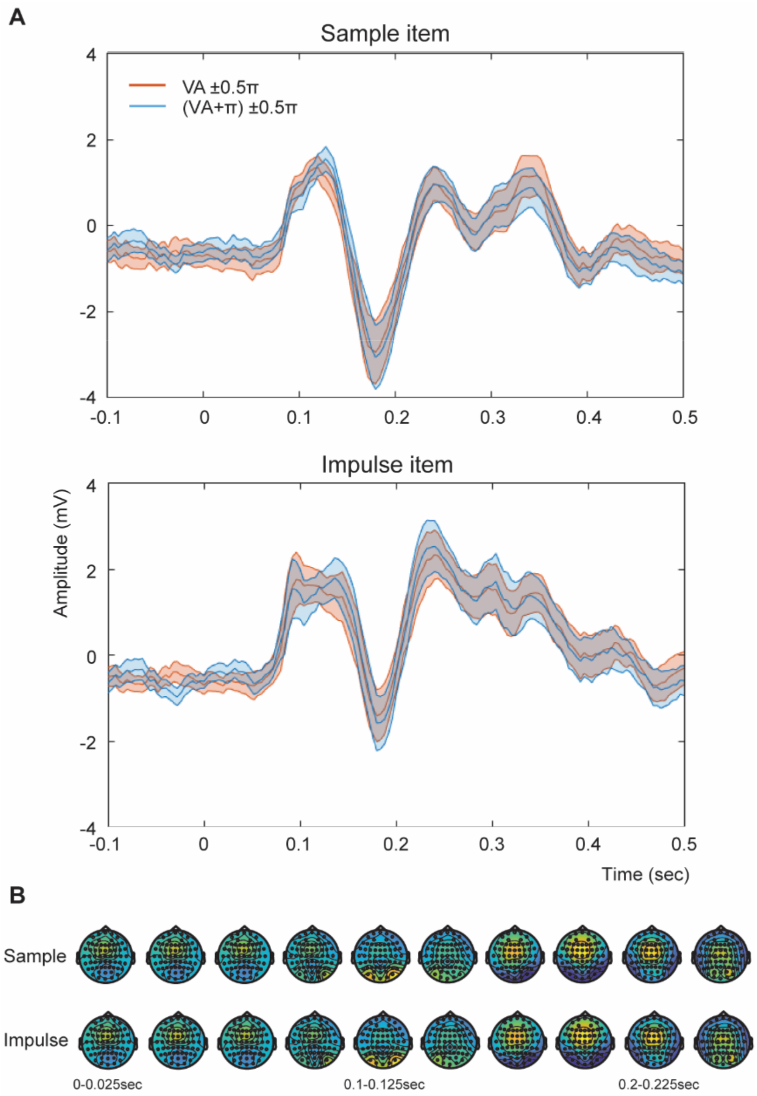

Supplementary Figure 5. Event related potentials (ERPs). A) ERPs of the sample (top) and impulse (bottom) stimulus for trials around +/- 0.5 π of the phase of the individual vector (blue) and around +/- 0.5 π of the phase of the individual vector + π (orange). Shaded areas represent the standard error of the mean (n=19). B) topographies of the ERP within -3.5 to 3.5 mV.

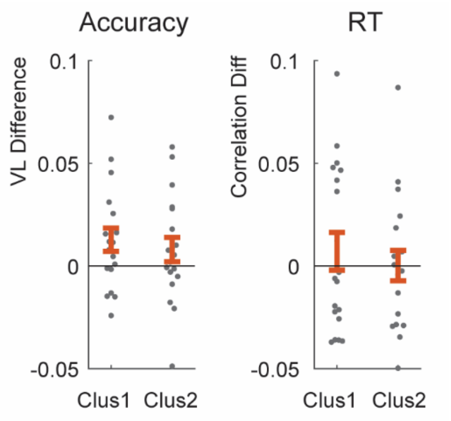

Supplementary Figure 6. Behavioral effects for the two clusters separately. Error bars represent the standard error of the mean (n=19). No difference was found between the two clusters. No effect of reaction time (RT) was found.

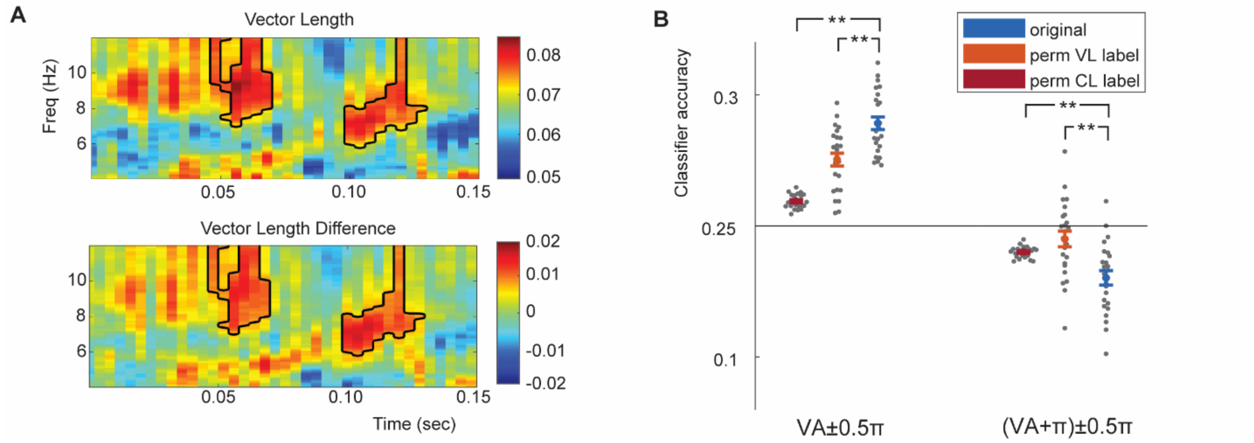

Supplementary Figure 7. Phase-dependent decoding of working memory content by a classifier trained on the sample item for the dataset of Wolff et al. Error bars represent the standard error of the mean (n=24). Conventions are the same as in Figure 1.

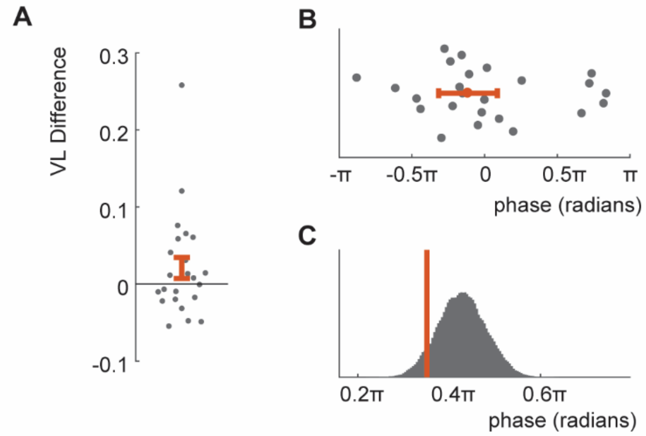

Supplementary Figure 8. Relation between behavior and oscillatory phase at impulse onset. Error bars represent the standard error of the mean (n=24; in B it represents the circular variance). Conventions are the same as in Figure 2.

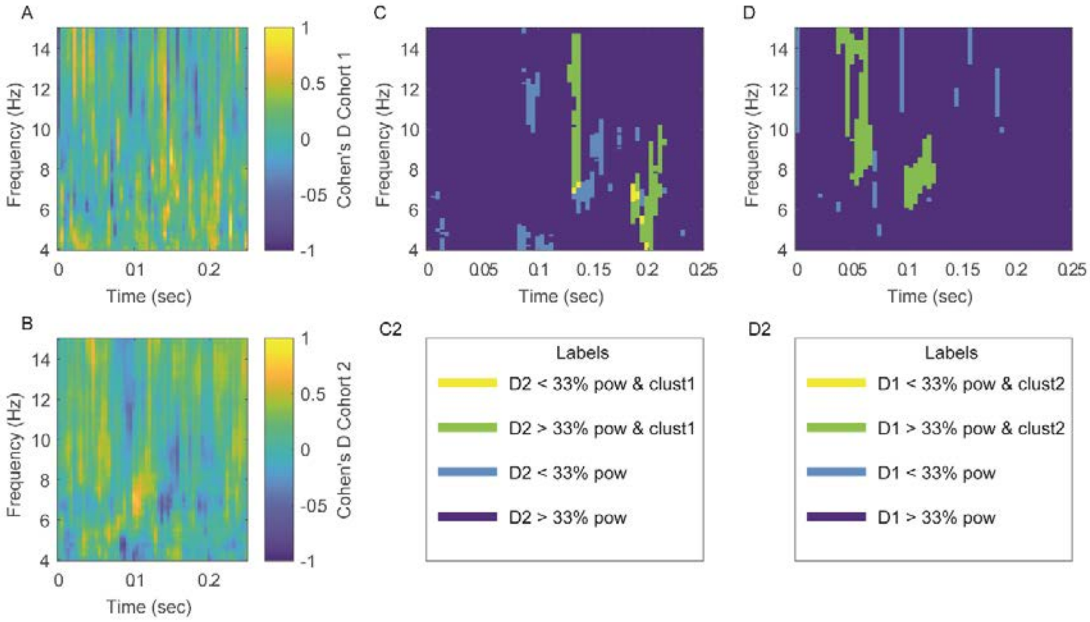

Supplementary Figure 9. Evaluation of replication results. A) Cohen's D for cohort 1 (our data). B) Cohen's D for cohort 2 (Wolff's et al.,[1] data). C) Power comparisons to determine whether there was evidence that Cohen's D of cohort 2 was lower than what would be detected at a power of 33% in cohort 1. Different colors indicate the following: *Dark blue*: Cohen's D of cohort 2 was not significantly lower than what would be detected at a power of 33% in cohort 1 outside of the clusters of cohort 1 (D2>33%pow). *Light blue*: Cohen's D of cohort 2 is significantly lower than what would be detected at a power of 33% in cohort 1 outside of the cluster of cohort 1 (D2<33%pow). *Green*: Cohen's D of cohort 2 was not significantly lower than what would be detected at a power of 33% in cohort 1 and is within the cluster of cohort 1 (D2>33%pow & clust1). *Yellow*: Cohen's D of cohort 1 was significantly lower than what would be detected at a power of 33% in cohort 1 and is within the cluster of cohort 1 (D2<33%pow & clust1). Note that light blue points were never significant in the original dataset, and therefore were irrelevant for judging the replication success for a significant effect. D) Same as C but investigating whether there was evidence that Cohen's D of cohort 1 was lower than what would be detected at a power of 33% at cohort 2.

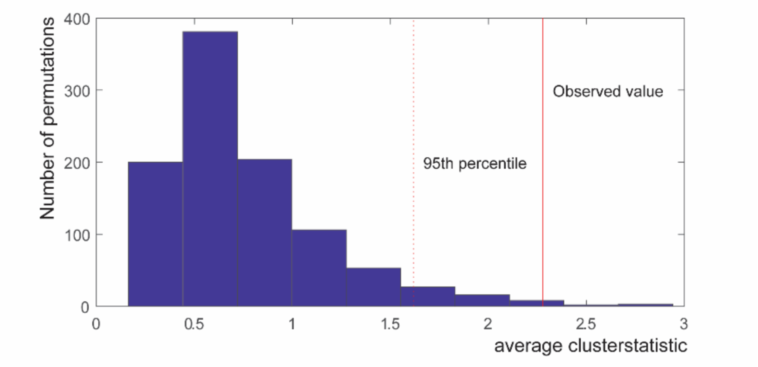

Supplementary Figure 10. Histogram of the null distribution for the average over two cohorts. Dotted and full red line indicate the 95th percentile and observed average cluster statistic.

Supplementary Note 1

The main analysis of this study was repeated for an available dataset from a study from Wolff et al[1]. Our design was inspired by their study but we made some modifications. We had a more difficult task, including orientation differences of 2 degrees, and we made the timing of the impulse stimulus less predictable. Furthermore, their study did not include a long interval no-impulse trials, which made behavioural comparisons to the no-impulse trial not possible.

Phase dependent MVPA decoding: decoding performance of the MVPA was modulated by phase in the dataset of Wolff et al.[1] (Supplementary Figure 7; Comparable to Figure 1A). Two significant clusters were found: 1) a cluster ranging from 7-12 Hz and 0.048-0.068 sec (cluster statistics = 2.07, p = 0.035) and 2) a cluster ranging from 5.8-12 Hz and 0.1-0.128 sec (cluster statistics = 2.00, p = 0.038). While the cluster's frequency ranges were very similar, the timing of the two clusters seemed to be earlier than in our own dataset. However, further analysis comparing effect sizes as a function of time between the two datasets revealed a compatible time course of effect sizes in the two datasets (Supplementary Figures 9 and 10). More importantly, just like in our data (Figure 1B), the data of Wolff et al.[1] showed higher decoding performance for the VA phase bin when compared to both the permuted classification accuracy labels ($t(23) = 6.9525$, $p < 0.001$), and the permuted orientation labels ($t(23) = 12.298$, $p < 0.001$). The VA + π phase bin showed significantly lower decoding performance for both phase bins (($t(23) = -7.1392$, $p < 0.001$ for the comparison with permuted classification accuracy labels and $t(23) = -3.5431$, $p = 0.002$ for the comparison with permuted orientation labels).

Phase dependent behavioural performance: There was a trend for the behavioural vector length modulation (Supplementary Figure 8; $t = 1.5407$, $p = 0.069$) such that accuracy was modulated by phase, similar as in our results (Figure 2A). Moreover, the phase difference between the vector angle of the phase modulation and the vector angle of the decoding performance modulations was not significantly different from zero ($Z = 8.249$, $p = 0.009$; Figure 2C).

Supplementary Note 2

To test whether there was any evidence for inconsistency on a datapoint-by-datapoint basis between the dataset of Wolff et al.,[1] and our own dataset we followed an approach by Simonsohn[2]. In this approach, one investigates whether the effect size in the replication cohort is statistically different from an effect size at which an effect was detectable in the original dataset. This constitutes a test of replicability. We followed Simonsohn's[2] proposal to investigate effect sizes at a

power of 33% (i.e. a small effect) in the original dataset (cohort 1, our dataset). Effect sizes were estimated using Cohen's D (the standardized difference between means). Only sporadic datapoints at the edge of the cluster, indicated in yellow in Supplementary Figure 9C, showed Cohen's D in cohort 2 (dataset of Wolff et al.,[1]) that were significantly lower than detectable at an effect size of 33% in cohort 1. Reversing the order of the test (comparing Cohen's D of cohort 1 at an effect size at 33% power detectable in cohort 2) did not yield any point at which Cohen's D was lower within the clusters of cohort 2. Therefore, on a datapoint-by-datapoint basis there was no evidence that the data in the two datasets are inconsistent with each other.

In another approach to assess reproducibility of our main inferential statistic over the two dataset (the cluster statistic) we extracted the probability under the null hypothesis of no cluster in the data given the information of both datasets. As such, we created a new test statistic: the average of the cluster statistics from both cohorts. The null distribution under this null hypothesis is constructed by creating a null distribution of the average of the random permutations. The p-value can then be extracted by the number of permutations that have a cluster statistic higher than this observed value (expressed as a proportion of the total number of permutations). The p-value in this approach is 0.007, thus rejecting the null-hypothesis that for the combined data there would be no significant cluster (Supplementary Figure 10).

Supplementary Discussion

The exact timing of phase-dependent decoding in the dataset from Wolff et al. appeared earlier compared to our dataset (0.05 and 0.1 sec in Wolff's dataset compared to 0.15 and 0.20 sec in our dataset). There are small differences in experimental design (e.g., in the predictability of the impulse stimulus which was jittered between 1100-1300ms in our study versus 1170 - 1230ms in Wolff et al.) that could have caused small variations in analysis outcomes between Wolff et al.'s study and ours. However, note that the windows of enhanced decoding are within a time range following the impulse stimulus within which sensory effects of the impulse stimulus would have their maximal effect. Hence the effects in both studies occur in acceptable time windows following the impulse stimulus. Moreover, a statistical comparison between the datasets following Simonsohn's proposal to investigate effect sizes did not support the idea that the two datasets were inconsistent with respect to the timing of the clusters (Supplementary Figures 9 and 10). This means that the exact timing of the clusters as shown in Figure 1 and Supplementary Figures 7 and 9 does not carry

enough wait to warrant any interpretation. This is also in line with the notion that no inferences can be made about the exact location of a time/frequency cluster[3,4].

The exact origin of the phase-dependent distributed response to the impulse stimulus is at present unclear, but we can conceive of two broad categories of mechanisms. On the one hand, the phase-dependent distributed response could result from differences in the evoked response among electrodes, or, alternatively, it could result from a cross-talk between the impulse-evoked responses at the different electrodes and the ongoing oscillatory brain activity. According to the former idea, from trial to trial, the impulse will hit ongoing oscillations and induce a phase-reset, aligning all phases to one phase (i.e. the post-impulse decoding is independent from ongoing oscillatory activity). For impulse presentations at specific phase ranges, the evoked responses would more clearly resemble the evoked response elicited by the original sample stimulus, resulting in an increased VL. One mechanism by which this might occur is that, too the extent that WM content is encoded in small differences in activity of neuronal population at specific phase ranges, the strong phase-reset would momentarily reveal these amplitude differences among neuronal populations more clearly, and make it possible to read them out from differences in EEG scalp topographies. Thus, the phase-reset is in this case paired with a magnification of relative differences in amplitude among channels, leading to enhanced decoding of WM content a little while after impulse presentation. Alternatively, according to the latter idea, it is possible that part of the ongoing oscillatory signals and their phases are preserved after the impulse stimulus so that they interact with the evoked responses, in a manner that manifests itself in phase-dependent decoding of WM content following the impulse stimulus. While we did not find any evidence for increased decoding at specific phase ranges in the retention period prior to the impulse, it is still possible that the preservation of ongoing oscillations after the impulse stimulus adds to the decoding performance. At this moment it is difficult to decide among these and likely also other possible mechanisms.

Supplementary References

1 Wolff, M. J., Ding, J., Myers, N. E. & Stokes, M. G. Revealing hidden states in visual working memory using electroencephalography. *Frontiers in systems neuroscience* **9**, 123 (2015).

2 Simonsohn, U. Small telescopes: Detectability and the evaluation of replication results. *Psychological science* **26**, 559-569 (2015).

3 Maris, E. & Oostenveld, R. Nonparametric statistical testing of EEG-and MEG-data. *Journal of neuroscience methods* **164**, 177-190 (2007).

4 Sassenhagen, J. & Draschkow, D. Cluster-based permutation tests of MEG/EEG data do not establish significance of effect latency or location. *Psychophysiology*, e13335 (2019).
