# Supplementary material for: Phase-dependent amplification of working memory content and performance
Source: Nat Commun. 2020 Apr 14;11:1832. doi: 10.1038/s41467-020-15629-7 (PMC7156664; doi:10.1038/s41467-020-15629-7)
Supplement: Supplementary file 3 — Reporting Summary [file 41467_2020_15629_MOESM3_ESM.pdf]

nature research

Corresponding author(s): Sanne ten Oever

Last updated by author(s): 9-3-2020

Reporting Summary

Nature Research wishes to improve the reproducibility of the work that we publish. This form provides structure for consistency and transparency in reporting. For further information on Nature Research policies, see Authors & Referees and the Editorial Policy Checklist .

Statistics

For all statistical analyses, confirm that the following items are present in the figure legend, table legend, main text, or Methods section.

n/a	Confirmed	
☐	☒	The exact sample size (n) for each experimental group/condition, given as a discrete number and unit of measurement
☐	☒	A statement on whether measurements were taken from distinct samples or whether the same sample was measured repeatedly
☐	☒	The statistical test(s) used AND whether they are one- or two-sided<br>*Only common tests should be described solely by name; describe more complex techniques in the Methods section.*
☒	☐	A description of all covariates tested
☐	☒	A description of any assumptions or corrections, such as tests of normality and adjustment for multiple comparisons
☐	☒	A full description of the statistical parameters including central tendency (e.g. means) or other basic estimates (e.g. regression coefficient) AND variation (e.g. standard deviation) or associated estimates of uncertainty (e.g. confidence intervals)
☐	☒	For null hypothesis testing, the test statistic (e.g. F, t, r) with confidence intervals, effect sizes, degrees of freedom and P value noted<br>*Give P values as exact values whenever suitable.*
☒	☐	For Bayesian analysis, information on the choice of priors and Markov chain Monte Carlo settings
☒	☐	For hierarchical and complex designs, identification of the appropriate level for tests and full reporting of outcomes
☒	☐	Estimates of effect sizes (e.g. Cohen's d, Pearson's r), indicating how they were calculated

Our web collection on statistics for biologists contains articles on many of the points above.

Software and code

Policy information about availability of computer code

Data collection	Data collection was performed using Presentation software (20.0) and BrainVision Recorder (1.22).
Data analysis	Data was analyzed using Matlab 2017a. Open source software used were: Fieldtrip (version 20161231), EEGlab (14_1_1_b), the circular toolbox (2012a), Cosmo (2016), modelfree (version 1.1), and custom Matlab scripts.

For manuscripts utilizing custom algorithms or software that are central to the research but not yet described in published literature, software must be made available to editors/reviewers. We strongly encourage code deposition in a community repository (e.g. GitHub). See the Nature Research guidelines for submitting code & software for further information.

Data

Policy information about availability of data

All manuscripts must include a data availability statement. This statement should provide the following information, where applicable:

- Accession codes, unique identifiers, or web links for publicly available datasets
- A list of figures that have associated raw data
- A description of any restrictions on data availability

The EEG data and behavioral logfiles related to the main figures are available in the repository of Maastricht University [lin]

Field-specific reporting

Please select the one below that is the best fit for your research. If you are not sure, read the appropriate sections before making your selection.

☐ Life sciences ☒ Behavioural & social sciences ☐ Ecological, evolutionary & environmental sciences

Behavioural & social sciences study design

All studies must disclose on these points even when the disclosure is negative.

Study description	The study is a human EEG study including EEG data as well as behavioral data (accuracy and reaction times). All data are quantitative.
Research sample	We collected a representative sample of total 20 participants completed the experiment (mean age: 24.4, range 18-45, 15 females). The sample is representative as we were investigating the fundamental brain operations.
Sampling strategy	Random sampling. However, the advertisement was limited within a student population. The sample size was determined based on the sample size of similar previous studies (see e.g. Ten Oever & Sack, 2015; Ten Oever et al., 2017). This is representative as we are trying to find similar effect sizes as these studies.
Data collection	Data was collected in a soundproof room using BrainProducts EEG equipment and computerized tasks. The experimenter as well as the participant was present. The researcher was not blind to the experimental conditions. However, conditions varied trial-by-trial without the interference of the researcher.
Timing	30-04-3018 until 19-06-2018
Data exclusions	One participant was excluded due to low behavioural performance.
Non-participation	No drop-out
Randomization	All participants went through all conditions.

Reporting for specific materials, systems and methods

We require information from authors about some types of materials, experimental systems and methods used in many studies. Here, indicate whether each material, system or method listed is relevant to your study. If you are not sure if a list item applies to your research, read the appropriate section before selecting a response.

Materials & experimental systems

n/a	Involved in the study	
☒	☐	Antibodies
☒	☐	Eukaryotic cell lines
☒	☐	Palaeontology
☒	☐	Animals and other organisms
☐	☒	Human research participants
☒	☐	Clinical data

Methods

n/a	Involved in the study	
☒	☐	ChIP-seq
☒	☐	Flow cytometry
☒	☐	MRI-based neuroimaging

Human research participants

Policy information about studies involving human research participants

Population characteristics	see above
Recruitment	Recruitment was done via advertisement within a student population. This is a limited population and might restrict the generalizability over a wider age and socio-economical background. All students within this population are required to participate in 20 hours of research during their studies, possible limiting self-selection bias. However, this bias cannot be fully excluded and might limit generalizability as students are free to choose which experiment to participate in.
Ethics oversight	The local ethical committee of the faculty of Psychology and Neuroscience at Maastricht University.

Note that full information on the approval of the study protocol must also be provided in the manuscript.
